# Dysphagia in Frail Patients Is Not Frailty Dysphagia

**DOI:** 10.3390/geriatrics3040082

**Published:** 2018-11-19

**Authors:** David G. Smithard

**Affiliations:** 1 Queen Elizabeth Hospital, Lewisham and Greenwich Trust, Woolwich, Greater London SE18 4 QH, UK; david.smithard@nhs.net; Tel.: +44-20-8836-6000; 2 Department of Sports Science, University of Greenwich, London SE10 9LS, UK

**Keywords:** dysphagia, frailty, ageing, swallow

Society revolves around food, both as a physical necessity and a social nicety; thus, eating and drinking (and, hence, swallowing safely) have become a cornerstone of social life. Those for whom eating, drinking, and swallowing are so difficult that “normal” (standard-consistency and culturally acceptable) food cannot be eaten, may incur isolation [1]. More people are living to a great age (>85 years), and, over the next 30 years, those aged >65 years will constitute 30% of the population.

Of those over 85 years of age, 24% will be frail, and, as the population and people age, the absolute number of very old and frail people will increase.

Although the actual mechanics of swallowing do change with increasing age, swallowing remains safe. Recent work has suggested that the built-in safety of the swallow is due to an increased amount of variability in an aged individual’s swallowing process compared to younger people [2], rather than to fixed pairings of events [3]. This variability may enable subtle unconscious compensatory changes to occur when an otherwise well older person has problems swallowing [4]. These will result in pharyngeal pressure changes, reduced stripping wave, lower laryngeal elevation and reduced anterior movement, shortened upper oesphageal sphincter (UES) opening, and increased pharyngeal residue and bolus dwell time. Understanding the muscular changes in the supra-hyoid and pharyngeal muscles, including pressure changes within the pharynx and UES, is important to understand what is normal and prevent over-medicalisation of an otherwise normal swallow [5].

Dysphagia is more common in older people (>85 years versus <45 years), but the actual frequency in otherwise well older adults is hard to determine because of the under-reporting of swallowing problems, under-recognition, and poor study designs. Swallow problems may go unrecognised because of subtle and gradual changes in the swallow pattern, food consistency, or amounts eaten due to fatigue. Care facilities including hospitals do not routinely screen or proactively look for swallowing problems in frail older people (whereas this occurs for stroke patients). Perhaps, routine screening of those aged >85 years should occur, considering the frequency of dysphagia in this age group on acute admission to hospitals.

There are many underlying medical conditions within which dysphagia may occur, and, being associated with physical decline and poor outcome, dysphagia is a Geriatric Giant. Frailty, sarcopenia [6], dysphagia, and polypharmacy (and, hence, a high anticholinergic burden) are common bedfellows, each one influencing outcomes, but are they synergistic or complementary? Do they work independently or, when combined, produce effect amplification? 

Frail older people are at a physiological tipping point such that even a minor illness may put undue stress on their already fragile systems, including on the ability to swallow safely. With the removal of the stressing agent (e.g., medication changes, clinical improvement or recovery) the swallow should improve [7]. 

Death is a certainty, and its proximity makes it more so for frail older people. As the end of life nears, the approach to managing eating, drinking, and swallowing needs to change. An aggressive management is generally not appropriate, and neither is inactivity. There needs to be proactive and decisive decision-making in the management of swallowing and dysphagia to provide all parties (staff, patient, family) with a clear, unambiguous pathway of care. This may include no food and drink orally, feeding at risk, or enteral feeding. The terminology used is frequently confusing and variable. 

There remains a lot to be discovered and understood about swallowing in frail older people. People are not (any more) old at 65 years, therefore, researchers should stop suggesting that they are. We need to understand the swallow in frail older people living in the community, so that we can define what is acceptable and normal and avoid developing interventions that are not required and may be harmful.

## References

[1] Farpour S., Farpour H.R., Smithard D., Kardeh B., Ghazaei F., Zafarghasempour M. (2018). Dysphagia management in Iran: Knowledge, attitude and practice of health care providers. Dysphagia.

[2] Hertberg E.G., Lazarus C., Steele C.M., Molfenter S.M. (2018). Swallow event sequencing: Comparing healthy older and younger adults. Dysphagia.

[3] Kendall K.A., Leonard R.J., McKenzie S.W. (2003). Sequence variability during hypopharyngeal bolus transit. Dysphagia.

[4] Cock C., Omari T. (2018). Systematic Review of pharyngeal and esophageal manometry in healthy or dysphagic older persons (>60 years). Geriatrics.

[5] Chen P.H., Golub J.S., Hapner E.R., Johns M.M. (2009). Prevalence of perceived dysphagia and quality-of-life impairment in a geriatric population. Dysphagia.

[6] Cichero J.A.Y. (2018). Age-related changes to eating and swallowing impact frailty: Aspiration, choking risk, modified food texture and autonomy of choice. Geriatrics.

[7] Morris J.S., Hollwey F., Hansjee D., Power R.A., Griffith R., Longmore T., Smithard D.G., Dann-Reid E., Wright D.J. (2018). Pilot of a Charter to Improve Management of Medicines and Oral Care for Residents with Dysphagia in Care Homes. Geriatrics.

